# Hospital and Institutional News

**Published:** 1914-05-02

**Authors:** 


### HOSPITAL AND INSTITUTIONAL NEWS.

### HOSPITAL PREPARATIONS IN BELFAST.

The gun-running incident, which has caused so much excitement to politicians, brings clearly into prominence the preparations that have been made in Belfast and Ulster to meet possible emergency calls upon the hospitals. These preparations, which may be divided into two parts—those which the voluntary hospitals have undertaken and those which have been improvised by the volunteers and their friends—have had the preliminary test of the hard work which the tension of the past few weeks has accentuated. At the Royal Victoria Hospital, Belfast, no special provision for eventualities has been made beyond the preparation of two wards with sixteen beds in each, which are now ready for immediate occupation should they be needed. As a matter of fact, the committee and Lieut.-Colonel Deane, the superintendent, could hardly do more than this, for while the approximate number of available beds (out of 300) approaches 250, there is, as in many hospitals, really not sufficient staff to nurse more than these. Supposing hostilities occurred, many of the now occupied beds could be emptied, and such relatively minor operations as those for hernia would have to wait while the crisis continued. Moreover, the outbreak of civil war would mean, of course, the suspension of ordinary traffic and business, with the result that the daily tale of accidents incidental thereto would decline. It is therefore estimated that at urgent need about 200 beds in the Royal Victoria Hospital could be placed at the disposal of the troops.

### THE VOLUNTEER HOSPITALS IN ULSTER.

The hospital preparations of the Ulster Volunteer Force are on an extensive scale. A Medical Board has been established in the old Town Hall, Victoria Street, of which Lieut.-Colonel R. Davis, I.M.S., retired, is honorary secretary, while Dr. William Gibson is the honorary treasurer. Hospitals are being organised throughout the province by the aid of various medical men acting under the Board. The main feature of the plan, which we hope to describe in detail next week, is that each county is placed under a county director, who, working through the Medical Board, organises his division. Neither medical men nor the lay public in England have any adequate idea of the scale on which the preparations have been carried out. For instance, merely as a symbol of the careful details of the administration involved, there is a publication department, to which, of course, all information proceeding from the head office must be submitted. As we have pointed out already, the one permanent good and valuable lesson which the Ulster crisis will leave to hospital men is the example which it provides of the way in which emergency hospitals and Red Cross preparations in time of peace can be prepared, carried out, organised, and administered. Ambulance work has sometimes tended to degenerate into an outdoor game for the summer months. In Ulster it is a matter-of-fact reality, a true instance of the business of war, which is worth a dozen years of manœuvres and voluntary make-shifts.

### THE COMING CONFERENCE AT NEWCASTLE.

The fifth annual conference of the British Hospitals Association, as previously foreshadowed in our columns, will be held at Newcastle-on-Tyne from June 18 to 20. The presidential address will be delivered by Councillor Johnstone Wallace, Lord Mayor of the city, and the subjects of the papers, which, as usual, will be followed by discussions, are now in course of arrangement. Applications for membership and renewals of subscriptions should be addressed to the Hon. Treasurers, Mr. E. Stewart Johnson and Mr. R. A. Owthwaite, at the offices of the Association, 14 Victoria Street, S.W. The annual subscription is half a guinea, and, as our readers know from the full reports of the proceedings which regularly appear in The Hospital, every hospital manager who attends becomes a recruiting centre for the next conference, from the knowledge he has gained by personal intercourse, quite apart from the pleasure of the meetings and social events. All communications should be addressed to Mr. Conrad W. Thies, or to Mr. Alexander Hayes, the Honorary Secretaries, at 14 Victoria Street, Westminster. According to the provisional arrangements, Mr. P. J. Michelli, C.M.G., secretary of the *Dreadnought* Seamen's Hospital, Greenwich, will read a paper on "Pensions for Hospital Officers." Dr. D. J. Mackintosh, M.V.O., Chairman of the Council of the Association, is securing a paper from Glasgow, probably by one of the staff of the Western Infirmary, of which he is superintendent, and Mr. Roden H. P. Orde, house governor of the Royal Victoria Infirmary at Newcastle, to whom has been entrusted the arduous duties of honorary local secretary, is arranging for two papers from members of his infirmary staff. Full particulars will probably be available for publication in our next issue.

### HONOUR WHERE IT IS DUE.

With a characteristic disregard of convention, Sir Ronald Ross has addressed to the Chancellor of the Exchequer a petition for some recognition by Parliament of his discoveries in regard to the propagation of malaria. Unaccustomed as scientists are to adequate monetary rewards for their labours, this bold step by Sir Ronald may seem to many of them undignified. Yet there is much to be said in favour of the principle he upholds, and of its application to his own particular case. England has been much too backward in acknowledging the services to humanity at large, and to the material prosperity of her own tropical dependencies, of the band of self-sacrificing investigators whose researches have won them worldwide fame. Both Sir Patrick Manson and Sir Ronald Ross, the admitted leaders (with Sir

David Bruce) of this splendid work, had to wait far too long for the titular distinctions which are now honoured by association with their names. If these three (and many others in lesser degree) have made their names immortal, that is all the more reason why their country should see to the material side of their due reward. Should Parliament see fit to accede to Sir Ronald Ross's petition, his two fellow-workers cannot in justice be passed over; and we hope that all three will receive substantial grants. We have always held that in the matter of decorations, too, the British School of Tropical Medicine has not had its due; and that the doyen of the school should be admitted to the Order of Merit whilst this great veteran still flourishes in our midst.

### SALVARSAN IN POOR-LAW INFIRMARIES.

Dr. C. Thackray Parsons, medical superintendent of Fulham Infirmary, gave some interesting evidence before the Royal Commission on Venereal Diseases at its thirty-second meeting last week. He stated that salvarsan or neo-salvarsan had been used at the infirmary since April 1911, and that the results had been very striking in cases of acquired syphilis, less successful in congenital cases, and only of temporary alleviation in para-syphilitic conditions. He added that while neo-salvarsan had been better tolerated, it had been also less efficacious than salvarsan itself. It appears also from Dr. Thackray Parsons' evidence that patients in Poor-Law infirmaries have need of a good deal of persuasion in some cases before consenting to be submitted to the treatment, and that, even when their consent has been obtained, it is not always easy to induce them to remain until it is completed. We would refer our readers to the leading article on "The Treatment of Syphilis in Poor-Law Infirmaries," which will be found on the first page of this issue. It deals with the problem as a whole, and outlines a policy and reforms that, far-reaching as they are, will, we believe, be supported warmly by Poor-Law medical officers and—we hope in time—by Mr. Samuel himself.

### WINSLEY SANATORIUM AND THE CHARITY COMMISSIONERS.

At a recent meeting of the board of management of Winsley Sanatorium it was stated by the house committee that all the thirty additional patients' beds are fully equipped, and most of them let temporarily to local Insurance Committees pending the settlement of the constitution in accordance with the demands of the Local Government Board and the Charity Commissioners. When a settlement has been concluded these beds are to be transferred to the Bristol and Bath Corporations and to the Wilts County Council. This means, states the house committee, that, directly the constitutional question is settled, £4,500 will be received in respect of these beds. The management is resisting the placing of the institution under the control of the Charity Commissioners, and is engaged in negotiations in the matter. Alderman G. Pearson presided, and explained that the Local Government Board would not allow Bath, Bristol, and Wilts to borrow money to pay for the beds which these authorities were under contract to purchase, nor would they justify their undertaking to pay for the maintenance of patients using these beds. The Local Government Board said in effect: "Unless this matter is put in a form we consider right, we will not permit the purchase or the maintenance." The Chairman described the request from the Charity Commissioners to send their documents of title up to that body, and the reply of the Commissioners was: "If the Local Government Board are satisfied with your representations that this institution is being properly worked, without assistance, we will withdraw any claim to you to put yourselves under us." The view of the Local Government Board was, he said: "We have doubts whether the institution can be carried on." The deputation replied: "It can." The Local Government Board said further: "If you can satisfy the legal department the scheme can be legally carried out, we will allow the various localities to borrow money for the purchase of beds and to pay the contributions for the maintenance of the persons occupying them." The Town Clerk of Bristol wrote to the Charity Commission, but they had no reply. The report of the house committee was approved.

### INTERESTING THE WORKPEOPLE.

We describe elsewhere in this issue an interesting gathering of Leicester workpeople, which Sir Edward Wood's hospitality as chairman of the Royal Infirmary brought about. Social functions of this kind, which bring the contributing workpeople into close touch with modern hospital construction, are of educational as well as financial value, and Sir Edward Wood is to be congratulated on the double interest which his hospitality is inspiring.

### BLACKBURN "ROYAL" INFIRMARY.

On Thursday, last week, the Secretary of the Blackburn and East Lancashire Infirmary, Mr. Nathan A. Smith, received a letter from the Home Secretary stating that to mark the jubilee of the institution the King had been pleased to command that in future the institution shall be known as the Blackburn and East Lancashire Royal Infirmary. On the occasion of the visit of the King and Queen to Blackburn last July, the Royal motor-car was slowed down when passing the Infirmary. Founded in 1858, by Mr. W. Pilkington, Mayor of Blackburn, the cost of the original building and site was £25,000. In 1893 the Nurses' Home was added. In 1897 the Victoria Wing formed the town's memorial of the Diamond Jubilee. The whole scheme, which was not completed till 1901, cost £11,000. In 1908 the wards and out-patients' department were extended, among other things, by the provision of an *x*-ray room. A centralised heating system in place of the one which has been in use since the institution was built is now wanted.

### THE DOCTORS' WIVES ASSOCIATION.

We have placed ourselves in communication with the whole of the doctors' wives who have provisionally joined this proposed association. We have given to each of those who have sent in their subscription the option of having it back or of joining us in going forward with the formation of the Doctors' Wives Association. The work will be thoroughly well organised and can be made more perfect in the early days of the movement, before the members become so numerous as to entail very heavy work in many directions. Whether the members are few or many, the advantages which will accrue to individual members who join the association will be numerous and valuable. Those proposed members who have not yet paid their subscription of 7s. 6d. should do so in the course of the next few days; and those who have sent remittances should intimate to us their wishes in regard to them without further delay. No member will incur any liability beyond the annual subscription, which will be payable in advance each year.

### NEW WING AT BRISTOL GENERAL HOSPITAL.

If expectations are realised, the new wing of the Bristol General Hospital will be ready for opening in June. The new wing, which is on the Redcliff side of the main block, comprises a female medical ward and a maternity ward, both of which are provided with balconies, a dental department, a reconstructed laundry, better accommodation both for the resident medical officers and the nursing staff. The internal arrangements, always seemingly the most time-requiring parts of a building scheme, are now being proceeded with, and it is upon the progress made with them that the fixing of the date for the formal opening ceremony depends. The new wing has cost some £45,000, and its completion will practically coincide with, or at any rate be the first important event in, Mr. T. W. Gregg's secretaryship, to which, our readers will recall, he was appointed quite recently, in succession to Mr. William Thywaites.

### SHOULD CHAIRMANSHIPS GO ROUND A COMMITTEE?

Mr. G. Shrewsbury Smith has been re-elected chairman of the Worcester General Infirmary. Two names were proposed for the vice-chairmanship—namely, Mr. R. Bruce Ward and Mr. Sydney T. Harris. The proposer of the latter contended that it was only fair that the office should go round the committee. Mr. Bruce Ward was, however, unanimously re-elected. In reply to the Dean of Worcester's question as to how the scheme of paying patients was working, he was told that so far there had never been more than two at a time. It was proposed by Mr. W. Curtler that a record be kept of the attendances of the members at committees and sub-committees, and the suggestion was accepted. The Chairman stated that the subscriptions and the general receipts showed a slight increase compared with those of the corresponding period last year. As regards the question of the office of chairman "going the round of the committee," and being, in fact, the perquisite of every committeeman in turn, the result of the voting may be taken to show the committee's disapproval of the principle. In small, inactive, and unimportant committees, where personal feelings are more important than efficient work, there may be no objection to it, but what intelligent man would accept any post on a committee of this kind? The truth is that a good committeeman need no more have the qualities of a good chairman than a good assistant secretary may have the administrative gifts required in the senior post. What are our readers' views?

### THE INFECTIOUS HOSPITAL GARDEN.

We have often discussed in The Hospital the advantages, under proper conditions, of the hospital garden, and contrasted the relative neglect which prevails in this country with the care expended upon the grounds of institutions on the Continent, and particularly in Germany. Sometimes, however, we garden better than we know, as appears from the report that the committee of the Surrey Smallpox Hospital at Clandon have discovered in their grounds a very scarce fungus, which has indulged its propensity for the roots of trees and shrubs to such an extent that a sum of £50 has been requisitioned, and granted for the purpose of destroying its growth. A private fence, it seems, has been so much infested by the fungus as to have required complete digging up and burning. Here is an example, indeed, of an infectious disease in shrubs having chosen, in Nature's spirit of irony, to attack the very trees of an institution which exists to control infectious disease in man!

### THE SEX QUESTION IN RED CROSS WORK.

The Duchess of Norfolk presided last week in London at the annual council meeting of the Sussex branch of the British Red Cross Society, during which Mr. E. A. Ridsdale, chairman of the executive committee of the central body, announced the promotion by the Society of an official magazine. In adding that the Society had become affiliated with the National Fire Brigades Union, he said that this latter step had helped to meet the demand for more men workers in Red Cross work. He remarked on the well-known preponderance of women in Red Cross activities, and stated that the Board of Trade now accepted the Society's certificate. The reason why women have predominated in Red Cross work has been, no doubt, because nursing has played such a large part in it, and that the importance of Red Cross nursing was first appreciated by a woman—Florence Nightingale, in fact—who was snubbed as an ignorant female for venturing to suggest the usefulness of trained women nurses in time of war. The fact remains, however, that such nursing as women are fitted for is rather the beginning than the end of Red Cross work, and this reminder of the opening for, and responsibilities of, the male sex in Red Cross undertakings is timely and useful.

### THE ROUTINE OF A "THREEPENNY" PRACTICE.

At an inquest last Saturday on the body of a child, Dr. Jelley, known in North-East London as "the threepenny doctor," in the course of his evidence became engaged in a contest of wits with one of the Coroner's jury. It appears that after Dr. Jelley had stated in his evidence that the cause of death was bronchial pneumonia a juryman asked: "Don't you think that when you were told in the early morning that the child was very ill you should have gone before 4 P.M.?" "Not at all," Dr. Jelley is reported to have replied, "I should not think of doing so unless a most urgent message were brought to me. If I answered all messages hurriedly like that I should be rushing all over London. . . . It would interfere with the routine of my practice. . . . Even if I had gone I could have done nothing. Forty-two years of professional experience has told me that in most cases attendance on people at the point of death is absolutely useless. . . . You cannot expect me to administer a cylinder of oxygen costing 7s. 6d. and only to get laughed at for my pains. Why, a few days ago I spent three-quarters of an hour trying to resuscitate a drowning person, and I never got any money for it." The Juryman: "Your work is purely a matter of business with you and nothing further?" Dr. Jelley's reply was "Certainly." On another juryman remarking that it was unfortunate that the child's mother did not see another doctor the "threepenny doctor" replied: "Yes, and that unfortunateness brought 200 people to my surgery after 6 P.M. last night for treatment." The honesty and frankness of the two speakers in this cross-examination, as it may be called, lets more public light into the conditions and routine of "threepenny" practice than is usually available outside the profession and the districts where it obtains. Our readers will have no difficulty in dividing the credit between the doctor and the jury for themselves.

### NEW ASSISTANT MEDICAL SUPERINTENDENT AT CAMBERWELL INFIRMARY.

Dr. J. W. Grice, M.R.C.S. (Eng.), L.R.C.P. (Lond.), has been appointed assistant medical superintendent at the Camberwell Infirmary and assistant medical officer at Gordon Road institution. Dr. Grice is at present senior resident medical officer at North Evington Infirmary, Leicester. He was previously clinical assistant at Melbourne Hospital, Australia, assistant medical officer at the Poplar and Stepney Sick Asylum, and clinical assistant at Guy's Hospital.

### WORDSWORTH AND A SMALLPOX HOSPITAL.

Two questions which come frequently before the public and excite the bitterest controversy are whether or not a huge price should be paid to keep a famous picture for the nation, and whether or not a particular building will, or will not, destroy the amenities of a place of natural beauty or historic interest. It is very difficult to keep a clear head in these controversies. A famous picture, if it happens to be the work of a great master, is priceless in the sense that it has no money equivalent, being merely worth whatever the ignorant public can be induced to pay under the sway of constantly changing fashions. There are no fashions in the price of bread or meat, so we arrive at a fair notion of the money-value which any particular sample will fetch. It is not so with works of art, owing to the influence of fashion, the essence of which is, in all departments, an absence of standard, training, and expert knowledge. Institutional officers are more concerned, however, with the opposition raised to the building of particular institutions on sites with artistic or historic associations. Thus a storm of opposition has been raised at the proposal of the Skipton Rural District Council to build an isolation hospital within a short distance of Waterford Ghyll, known to readers of Wordsworth's poetry from his "White Doe of Rylstone." Local authorities seem to possess such a malign genius for building ugly piles of brick disguised by the name of public institutions, that one always feels disposed to credit them with the worst intentions and the worst taste. At last Saturday's meeting, however, the Chairman, Mr. J. A. Slingsby, declared that the Council had no wish to spoil the scenery; had actually incurred damages for breaches of contract over two purchases for a site for the isolation hospital, and now intended to build at Sandy Beck Bar, unless a site near the present Winterburn Hospital on reasonable terms were offered. What are the Council's ideas of reasonable terms, on the one hand; and how far is the public protest sheltering prejudice against an isolation hospital in itself behind the great name of Wordsworth on the other?

### A HOSPITAL PRESIDENT'S RESIGNATION.

Sir Henry Grey, Bart., president of the Malvern Hospital, has tendered his resignation. In a public statement on the subject, Mr. W. D. Perrins, the chairman of the meeting at which it was made, said that the resignation was the outcome of a discussion in committee of certain internal questions relating to the management of the institution, in which, he regretted to say, Sir Henry did not see eye to eye with the committee. Mr. Perrins added that he was prepared to justify the action of the committee. Thereupon Mr. C. W. Dyson Perrins was elected president on the motion of Dr. H. E. Dixey. No election could be more satisfactory, since it is entirely due to Mr. Dyson Perrins' generosity that the present Malvern Hospital, over which much money and care have been spent, has been provided.

### QUEEN ALEXANDRA AND INFANT MORTALITY.

Pending the full development of the scheme for reconstructing the St. Katharine Hospital Foundation, which was described in The Hospital of March 7, p. 620, and of which a college for the training of women workers formed an important part, it is proposed to begin by appointing one or two health visitors in each of the East-end boroughs. On behalf of the Corporation a letter has been sent to the Mayors of Stepney, Bethnal

Green, Poplar, and Shoreditch, which inquires if one or more women health visitors, working under borough control, would not be welcome to visit infants between the ages of ten days and one year. Owing to the cost to the rates, the letter proceeds to point out, medical officers of health cannot secure sufficient health visitors, and, therefore, would presumably welcome such assistance as the new scheme can provide in this direction. Queen Alexandra is mentioned in the letter as learning that health visitors tend to reduce infant mortality and to prevent the physical deterioration of the mothers. In Shoreditch the infant mortality rate is 123 per 1,000—the highest of the four boroughs; in Bethnal Green, the lowest, it is " only " 96. The welcome so far accorded to the proposal shows that existing municipal health visitors welcome their reinforcement on every ground.

### THE CLERGY AND DEATH CERTIFICATES.

The Rev. J. F. Heyes has written a public letter from St. Barnabas' Vicarage, Bolton, supporting Sir Victor Horsley's argument for technical truth in death certificates, and makes a useful point by urging that the parochial clergy, who, he adds, know the urgency of the need almost as well as the medical profession itself, should co-operate in this direction. He also makes a suggestion that the special difficulty of libel actions, the loss of practice, offended relatives, and the like could all be overcome by the issue of two certificates. One, he urges, should be " the conventional open certificate as now issued for burial purposes," and the second a confidential, scientific, and sealed document " issued under a registered number known only to the practitioner and the registration authorities," which should be countersigned by the local medical officer of health. By this means, Mr. Heyes points out, the name need never be divulged except by order of the Courts and in the interests of justice. If Mr. Heyes is right in asserting that the parochial clergy are alive to the importance of technically true death certificates, then there can be no doubt that those of them who are institutional chaplains should take the lead in focussing the attention of their fellow-clergy to this proposal. The chaplains are the true intermediaries in this matter between instructed medical and uninstructed lay opinion; and the interest which has been aroused by our article last week in the *Institutional Worker* section of The Hospital leads us to think that Mr. Heyes should not be far wrong. What, at any rate, are the views of hospital chaplains on the matter, and how far are they sufficiently interested in vital statistics to be ready to help this attempt at reform?

### HOSPITAL EGG WEEK: MAY 12 TO 19.

This year Hospital Egg Week is to be held from May 12 to 19, and though The Hospital is, from the nature of the case, not a journal designed primarily for poultry-keepers, we trust that the announcement of this annual proceeding may catch the eyes of some of them, who by means of the Egg Week, if not in more intimate fashion, are brought into relation with the voluntary hospitals. Mr. F. Carl, 154 Fleet Street, once more invites poultry-keepers to send batches of new-laid eggs for the hospitals, for this the fifth year in succession. The eggs, it is scarcely necessary to add, should be packed very carefully and the carriage paid, while the boxes will be returned on request, for which purpose labels can be obtained on application. Three prize goblets are offered by the organisers, associated with the *Poultry World*, for the three largest collections of eggs, and a silver watch will be given for the biggest collection made by a child under twelve. If 30,000 eggs are obtained for the hospitals, last year's record will be broken, as we hope it may be in the interests of the patients' diet.

### UNALLOCATED SURPLUS FUNDS.

The unallocated surplus of some £200,000, over which there has been so many heart-searchings, is a stage nearer being distributed now that the London Insurance Committee has decided by twenty-seven votes to twenty-three that a scheme for its distribution be prepared and submitted to the Commissioners for approval. This £200,000 is the Panel Fund, and the number of doctors on the London panel is given as about 1,500. Mr. Danckwerts, K.C., has reiterated his opinion that the proposed distribution to the panel doctors, though advocated by the Chancellor of the Exchequer, would be illegal, and that members of a committee sanctioning it would be legally liable to make good the amount disbursed. Mr. Kingsley Wood's motion that the Commissioners be told that, in view of counsel's opinion, the committee could not distribute, was defeated, and Mrs. Handel Booth's amendment that a scheme be prepared and submitted to them was carried. We would specially refer our medical readers to the official pages at the end of The Hospital, in which the National Medical Union discusses the question and adumbrates a policy by drawing the moral of the impasse for the medical profession as a whole.

### MR. DANCKWERTS AND THE PROFESSION.

Since writing the paragraph immediately above, in this connection we must also record the sudden death of Mr. Danckwerts, the well-known K.C., whose opinion was sought and whose advice rejected so recently on this very matter. Only sixty-one years of age, and noted for his memory, his burly physique, and his trenchant and sometimes overbearing manner, Mr. Danckwerts was one of the personalities of the Bar, and the " tough nut " which he was recently asked to crack—and his penetrating acumen was nourished on such performances—gave him an interest to the hospital world that it is the lot of few King's Counsel, however eminent, to evoke.

### THE NEW WAYLAND INFIRMARY.

Some ten years ago, we believe, it began to stir the conscience of the Wayland Guardians that their clay-built institution at Rackland was out of date and medically impossible. As a result of an estimate of £4,000 for alterations being rejected by them as impracticable, in 1906 the Local Government Board architect, Mr. Kitchin, made an inspection, and virtually condemned the existing institution from top to bottom. The result was that in spite of opposition the new institution at Attleborough, which is in process of occupation, after further delays and difficulties, was built from the design of Mr. H. J. Green, of the neighbouring town of Norwich, at an estimated cost of £10,000.

### ITS LESSON FOR POOR-LAW HISTORY.

The story of the obstruction and delays resorted to by opponents of the new institution is a history in little of Poor-Law struggles everywhere, but it is useless to expect that the lesson will be taken home, even locally. The only thing which a study of history proves is that men learn nothing from it. Built with red-brick facings, the main block has a frontage of 250 feet, and the ground floor is devoted to the matron's and master's offices and apartments. The ends of the building are divided between the sexes, the upper floors being for the sick, and the lower for the infirm cases. Day rooms and verandahs are provided for the wards. Maternity wards occupy the first floor, together with a separation ward, surgery, and blanket stores. It is stated that the iron emergency stairs from the day-room corridor will be of much use to friends of patients on visiting days. The accommodation is for a hundred, in which no provision is made for a children's department.

### THE MISUSE OF THE TERM "INFIRMARY."

It is, we wish especially to remark, on the whole, a satisfactory sign of the times, as representing an aspiration, that the new workhouse, which is really what it is, should appropriate the title of "infirmary"—a name and style which strictly should be resumed for completely separated institutions. No great harm will be done by a technical misuse of the term infirmary, provided that the Wayland Guardians recognise that this title is a privilege to be earned and worked, and represents the next step in the progress of their institution. It may seem a long step ahead in view of the relative smallness of the institution at the present day, but that is no reason why the Guardians should not be alive to the fact that the problem of the sick in rural districts will probably be solved in the future by a centralised hospital for all the sick cases in the workhouses of each area. Once conscious of the trend of development half the past stupid opposition will probably escape repetition over again.

### THE POSITION OF MEXICAN DRUGS.

Little or no improvement has been noticeable in the drug market this week, and transactions appear to have been of a restricted character. Fluctuations in value either upwards or downwards have for the most part been of minor importance. There is practically no change in the position of citric acid, and no decline in price is looked for at present. There has been a slight advance in quotations for tartaric acid, and a further advance would not be surprising; the demand is good, as is usual at this season of the year. Cream of tartar is also rather dearer and in good demand. It is probable that no great advantage would be gained by delaying the purchase of such quantities of citric and tartaric acids and cream of tartar as may be required for use in the immediate future. There has been no further decline in quotations for cod-liver oil, and a fair amount of business has been done. Business has also been done in menthol, and the tone of the market is somewhat steadier. The price of essence of lemon is practically unchanged. Camphor is in slightly better demand. So far there has been no appreciable change in the position of Mexican drugs.

### THE CITY CORONER'S PRONOUNCEMENTS.

Dr. F. J. Waldo, the City Coroner, is one of those men who have the valuable power of making themselves heard, with the result that the City and Southwark Coroner's Courts probably provide mere "copy" for the lay press than any other in the Metropolis. It so happens that this activity of mind, accompanied as it is by a large amount of personal energy, has been turned, and valuably turned, in the direction in which the medical profession's influence can bear important fruit. The payment of medical witnesses in Coroners' Courts, the evidence before more than one Royal Commission are instances of good and strenuous work. The result is that when Dr. Waldo states a simple statistical fact, as, for instance, that certain months show a higher average of suicides than certain others, and paints this in a well-defined phrase, the lay press resounds with the discovery.

### THE REPORTING OF INQUESTS.

Our only complaint is directed not against Dr. Waldo, but against the editors who exploit his wit, and refuse to do their share of good and useful journalism, but stuffing behind it the more solid points which are made just as frequently. The fact remains that inquests are badly and slovenly reported, leaving us to feel that the editors of the dailies are still asleep to the fact that there is a mine of invaluable and unexploited "copy" in inquests altogether apart from their modest and unvarying elements of sensationalism.

### TO OUR READERS.

Contributions are specially invited from any of our readers to these columns. They should deal with topical subjects and news. They must be authenticated for the information of the Editor only. The minimum payment if published is 5s. These is no hard-and-fast rule as to space, but notes of about twenty lines in length are preferred.

